# Clinical efficacy and applicability of natural products in the treatment and prevention of radiotherapy-induced oral mucositis: A systematic review

**DOI:** 10.1371/journal.pone.0303988

**Published:** 2024-05-23

**Authors:** Wen Zhang, Lu Fan, Yifang Xie, Tenghui Gao, Jieping Zeng

**Affiliations:** Phase I Clinical Research Unit, Hospital of Chengdu University of Traditional Chinese Medicine, Chengdu, Sichuan, China; The University of the West Indies, TRINIDAD AND TOBAGO

## Abstract

The aim of this systematic review was to describe the efficacy and acceptability of natural products in the management of oral mucositis caused by radiation. From the day it started to August 7, 2023, a thorough search for randomized controlled trials (RCTs) was carried out among seven databases: the Web of Science, PubMed, Embase, OVID, Scopus, the Cochrane Library and the CINAHL database. Only English-language articles were identified during the search. Using the revised Cochrane risk-of-bias tool, version 2, two researchers screened the articles, collected information on study characteristics, and appraised risks of bias. The data were analyzed and descriptively presented with a narrative synthesis methodology involving the Synthesis Without Meta-Analysis (SWiM) reporting element applied in detail. The PROSPERO registration number of this study is CRD42023476932. Thirty-six clinical trials were included in the study; the included studies included a variety of 20 types of natural products. Honey and Curcuma longa were the most commonly assessed natural products. A total of 2,400 participants reported taking part in therapy with natural products for oral mucositis. Natural products demonstrated substantial efficacy in terms of influencing intensity, incidence, pain score, quality of life, and symptoms such as xerostomia and dysphagia. Except for manuka honey, most natural products were well accepted. Regarding the clinical trials’ risk of bias, 2 clinical trials (5.56%) had a high risk of bias, 17 studies (47.2%) had a low risk of bias, and 17 studies (47.2%) were rated with “some concern.” Natural remedies work well as alternate treatments for managing oral mucositis caused by radiation therapy. However, additional clinical trials are still needed. The safety of these conventional medications as well as their effectiveness and safety when used in combination with other conventional or naturopathic therapies should be fully examined.

## Introduction

The probability of developing mucositis is highly dependent on the sites irradiated. Patients who undergo routine radiotherapy (RT) fractionation for head and neck cancer have a greater probability of developing oral mucositis [1]. Radiation-induced mucositis can occur in up to 91% of patients with head and neck cancer who undergo radiotherapy [2]. In patients with head and neck cancer, oral mucositis is a common restricted cytotoxic lesion, and there may be incapacitating reactions caused by radiation exposure [3]. Radiation-induced mucositis manifests as dry mouth, dysphagia, severe erythematous ulcers, and many secondary infections [4]. Patients with severe oral mucositis may not be able to tolerate treatment, which could result in unwarranted hospital stays and treatment interruptions, impairing tumor control and patient survival and increasing the financial burden on society [5, 6].

Many strategies, such as oral hygiene, sodium bicarbonate, benzydamine hydrochloride rinses, glutamine, growth factor, cytokines, anti-inflammatory drugs, analgesics, low-level laser therapy, and cryotherapy [4, 7] have been suggested for treating oral mucositis, but none of these strategies are fully effective; moreover, some agents are associated with drug side effects and ancillary costs [8, 9]. In addition to being less expensive, more widely available [10], and having fewer side effects [11] than synthetic drugs, natural products also show potential in pharmacological laboratory studies and clinical trials for their antioxidant, anti-inflammatory, antimicrobial, immunomodulatory, and wound-healing properties [12], all of which may have varying degrees of impact on the pathophysiology of mucositis.

Based on the body of existing scientific research, the purpose of this systematic review was to evaluate the effectiveness, acceptability, and safety of natural products in the management of radiotherapy-induced oral mucositis.

## Methods

This systematic review was carried out in accordance with the Preferred Reporting Items for Systematic Reviews and Meta-Analysis 2020 statement [13]. This review was submitted to the National Institute for Health Research (PROSPERO) (CRD42023476932). We developed a population, intervention, comparison, and outcome (PICO) model.

### Search strategy

We systematically searched seven electronic databases, the Cochrane Library, Web of Science, PubMed, Embase, OVID, Scopus and CINAHL databases, from inception to August 7, 2023. The search terms included “oral mucositis”, “radiotherapy”, “natural products” and related synonyms. The detailed search strategies for each database were listed in S1 File. Two investigators independently searched for literature in seven databases. The third investigator verified the study selection process.

### Eligibility criteria

The inclusion criteria were as follows: (a) population—adults with a diagnosis of oral mucositis after radiotherapy or combination of radiotherapy and chemotherapy; (b) intervention—patients in treatment groups received a single natural product or the natural product in combination with another therapeutic strategy; and (c) control—patients in control groups received placebo, other standard agents, nothing or a combination of the above; interventions could be administered by any route, formulation or dose; (d) outcome—the primary outcome was the severity of oral mucositis (including, e.g., the incidence, score, even time of onset, duration), and acceptability of medicines; the secondary outcome was access to other symptom complications (e.g., pain level, quality of life assessment and others; e.g., xerostomia, dysphagia and cytokines).

The exclusion criteria were as follows: duplicates, no access to the full text, literature reviews, trial protocols, clinical studies without a control group, research conducted *in vitro* or animal, studies using a drug solution containing ethanol, and studies that could not verify the composition of specific constituents.

### Study selection and data extraction

Two authors eliminated irrelevant articles by reviewing the full title and abstract of each study based on the inclusion criteria. The eligibility of the articles was assessed by scanning the full texts of the articles that met the inclusion criteria, and detailed information on the study characteristics was independently extracted to data extraction forms. The extracted information was collected, including details about the author, study year, study design, participants, natural product of interventions, agent of comparators, agent administration route and outcome results. Disagreements were resolved by consensus or by consulting another researcher.

### Evaluation of the risk of bias

Two reviewers separately evaluated the risk of bias. Randomized controlled trials were assessed using the revised Cochrane Risk-of-bias Tool for Randomized Studies (RoB 2) [14]. Bias risk was divided into three categories: low, some concerns, and high.

### Methods for synthesis

Given the variation in interventions across study designs and lack of standardized data about the Consolidated Standards of Reporting Trials (CONSORT) statement on standards for herbal and Chinese medicine interventions, it was not feasible to perform a meta-analysis. The synthesis of findings was directed by narrative synthesis methods and the Synthesis Without Meta-Analysis (SWiM) reporting guidelines [15]. Synthesis descriptions were grouped according to outcome indicators.

## Results

### Study characteristics

**Fig 1:**
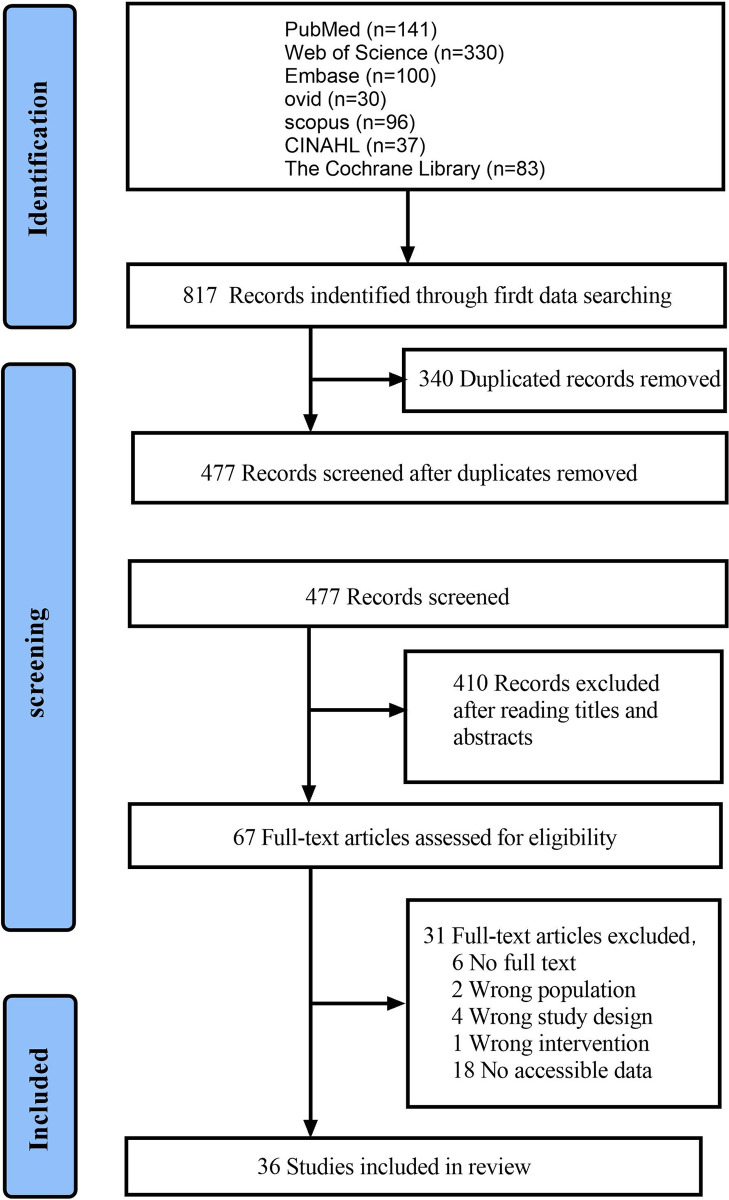
The flow diagram on the searching and screening.

**Fig 1** shows the process of database searching. A total of 817 studies were found. After completing the elimination of duplicate studies and assessing the titles and abstracts, we chose 69 studies for full-text evaluation. Finally, 36 studies that met the inclusion criteria were included in the qualitative analysis. **Table 1** generalizes the features of the studies included in this review, including information on study location, study design, funding, intervention duration and participant sample size. The included studies spanned 21 years (2003 [16] to 2023 [17, 18]). **Table 2** shows the fundamental characteristics of the clinical trials using natural agents. **Table 3** shows the main results of the primary and secondary outcomes of clinical trials included in the systematic review.

**Table 1:** Summary of clinical trials included.

Study Background Information	Study Design Details
Study location	Iran (10), India (7),
	China (6), Italy (2),
	Brazil (2), Canada (1),
	Turkey (2), Malaysia (1),
	Egypt (1), Cyprus (1),
	New Zealand (1), Germany (1),
	Taiwan (1), USA (1),
	Thailand (1).
Funding	University (12),
	Government (6),
	Pharmaceutical industry (4),
	No funding (3),
	Not mentioned (13).
Study design	All parallel design
Comparator	Placebo (23)
	Active intervention (8)
	Radiation combined with nothing (4) Conventional treatment with blank (1)
Duration of the trials	range from 6 weeks to 5months
Radiation dose	≥50Gy
Number of participants	Between 20 and 240

**Table 2:** Fundamental characteristics of included trials.

Author, Year (Country)	Study design	Sample size	Assesment time (week/day)	Intervention (Name of agent)	Route of administration (method)	Comparation	Assessment instrument
Biswal et al. 2003 (Malaysia) [16]	RCT	40	7 week (once a week)	pure honey from the tea plant (Camellia sinensis)	topical and systemic (rinse and swallow)	nothing	RTOG
Su, et al. 2004 (USA) [31]	double blind RCT	58	6 weeks (1^st^ week 3^rd^ week 6^th^ week)	Aloe Vera	Systemic (oral)	Placebo	RTOG UW-QOL
Naidu et al. 2005 (India) [44]	double blind RCT	30	2 weeks (once a week)	Mucotrol™ (MF 5232)	systemic (oral)	placebo	WHO RTOG
Wu et al. 2007 (China) [50]	RCT	60	6 weeks (1^st^ week 2^nd^ week 4^th^ week)	Qingre Liyan Decoction	systemic (oral)	Dobell’s solution	RTOG
Motallebnejad et al. 2008 (Iran) [25]	single blind RCT	40	6 weeks (once a week)	pure honey from Thymus and Astragale	topical and systemic (rinse and swallow)	normal saline	OMAS
Rashad et al. 2009 (Egypt) [27]	RCT	40	6 weeks (once a week)	pure honey from the clover plant Trifolium alexandrenum.	topical and systemic (rinse and swallow)	nothing	WHO
Maddocks-Jennings et al. 2009 (New Zealand) [37]	single blind RCT	26	5 weeks (once a week)	Essential oils (a 1:1 mix of maunka and kanuka oils)	topical (gargle)	Arm A: placebo Arm B: conventional treatment	RTOG
You et al. 2009 (Taiwan) [41]	RCT	20	7 weeks (1^st^ week 3^rd^ week 5^th^ week 7^th^ week)	Indigowood root	topical (gargle)	placebo	NCI-CTCAE v3.0
Putwatana et al. 2009 (Thailand) [32]	single blind RCT	60	7 weeks (once a week)	glycerin payayor	topical (gargle)	benzydamine	WHO
Jayachandran et al. 2012 (India) [26]	RCT	60	7 weeks (once a week) 2 weeks’ follow up	pure honey (Dabur honey)	topical and systemic (rinse and swallow)	arm A: benzydamine hydrochloride arm B: normal saline.	WHO
Pawar et al. 2013 (India) [46]	single blind RCT	30	7 weeks (once a week)	SAMITAL	topical (gargle)	placebo	WHO VAS UW-QOL
Hawley et al. 2014 (Canada) [28]	double blind RCT	106	6 weeks (once a week) 1 weeks’ follow up	manuka honey	topical and systemic (Swish and swallow)	Placebo	RTOG WHO OMAS
Sahebjamee et al. 2015 (Iran) [30]	triple blind RCT	26	7 weeks (once a week) 1 week of follow-up	aloe vera	topical (rinse)	benzydamine mouthwash	WHO UW-QOL
Mansourian et al. 2015 (Iran) [19]	Double blind RCT	37	7 weeks (once a week)	Curcuma Longa	topical (gel)	placebo	WHO VAS
Bolouri et al. 2015 (Iran) [38]	triple-blind RCT	20	5 weeks (once a week)	propolis	topical (gargle)	placebo	NCI-CTCAE v3.0
Elyasi et al. 2016 (Iran) [35]	double blind RCT	30	6 weeks (once a week)	silymarin	systemic (oral)	placebo	WHO NCI-CTCAE v4.0
Luo et al 2016 (China) [43]	RCT	240	6 weeks (once a week)	Kangfuxin Solution	topical (gargle)	Compound borax gargle	CTCAE v3.0 VRS
Marucci et al. 2017 (Italy) [47]	double-blind RCT	104	7 weeks (once a week)	Faringel (propolis, aloe vera, calendula, and chamomile)	topical (gargle)	placebo	NCI-CTCAE v3.0
Doğan et al. 2017 (Turkey) [34]	RCT	80	7 weeks (once a week)	black mulberry molasses	topical and systemic	nothing	NCI-CTCAE v4.0 OAG UW-QOL
Aghamohammadi et al. 2018 (Iran) [39]	double blind RCT	52	7 weeks (once a week)	zataria	topical (gargle)	placebo	OMAS WHO
Zheng et al. 2018 (China) [48]	double blind RCT	240	10days 20days 30days 40days 50days	Shuanghuabaihe Tablets	systemic (oral)	placebo	WHO CTCAE v3.0 OAG OMDQ
Charalambous et al. 2018 (Cyprus) [29]	RCT	72	7 weeks (once a week)	thyme honey	topical (gargle)	normal saline	RTOG
Wang et al. 2018 (China) [49]	RCT	70	7 weeks (once a week)	CHIN	systemic (oral)	Recombinant human epidermal growth factor (rhEGF)	CTCAE v4.0
Delavarian et al. 2019 (Iran) [21]	double blind RCT	32	4 weeks (once a week)	curcumin	systemic (oral)	placebo	NCI-CTCAE v2.0
Arun et al. 2020 (India) [23]	single blind RCT	64	4 weeks (once aweek) 2months’ follow up	curcumin	systemic (oral)	placebo	WHO NCI-CTCAE v2.0
Shah et al. 2020 (India) [20]	triple blind RCT	74	6 week (once a week)	curcumin	topical (mouthwash)	benzydamine mouthwash	WHO
Mamgain et al. 2020 (India) [52]	RCT	127	6 weeks (once a week)	conventional treatment + Glycyrrhiza glabra(Yashtimadhu)	systemic (oral)	arm A: conventional treatment arm B: conventional treatment +honey	RTOG
Soltani et al. 2020 (Iran) [42]	double blind RCT	46	7 weeks (once a week)	plantago major L	systemic (oral)	placebo	WHO VAS
Arantes et al. 2021 (Brazil) [24]	triple-blind RCT	62	7weeks (1^st^ week 2^nd^ week 7^th^ week)	FITOPROT	topical (gargle)	placebo	WHO NCI–CTVAE v4.0 UW-QOL VAS
Hosseini et al. 2021 (Iran) [36]	double blind RCT	38	6 weeks (once a week)	silymarin	systemic (oral)	placebo	NCI-CTCAE v4.0 EORTC
Ebert et al. 2021 (Germany) [40]	RCT	60	8 weeks (once a week and 19^th^ week 20^th^ week)	Cystus® tea	systemic (oral)	Sage tea	RTOG /EORTC
Soni et al. 2022 (India) [22]	double blind RCT	60	6 week (once a week)	turmeric extract	systemic (oral)	placebo	NCI-CTCAE v4.0
Fasanaro et al. 2022 (Italy) [45]	double blind RCT	120	7 weeks (once a week)	SAMITAL	topical (gargle)	placebo	OMAS WHO OAG XQ EORTCQLQC30 EORTCQLQ-H&N35
Sari et al. 2022 (Turkey) [33]	RCT	94	1^st^ week 3^rd^ week 6^th^ week	arm A: grape molasses arm B: black mulberry olasses	topical (gargle)	nothing	RTOG NCI-CTCAE v4.0 VAS EORTC QLQ-C30 EORTC QLQ-HN35
Martins et al. 2023 (Brazil) [18]	double blind RCT	52	6weeks (7^th^ day 14^th^ day 21^st^ day 30^th^ day)	FITOPROT+PBM+POCP	topical (gargle)	PBM+POCP	WHO NCI–CTVAE v5.0
Shirazian et al. 2023 (Iran) [17]	double blind RCT	30	8 weeks (once a week)	green tea	topical (gargle)	normal saline	WHO VAS

**Table 3:** Description of the primary and secondary outcomes of clinical trials included.

Author, Year (Country)	Intervention (Name of agent)	Results				
		data of oral mucositis	Pain	Quality of life	Acceptability and Adverse events	Others
Biswal et al. 2003 (Malaysia) [16]	pure honey from the tea plant (Camellia sinensis)	Grade: the honey group had lower incidence of severe oral mucositis(p = 0.00058).	NR	NR	Four patients (20%) was interrupted among controls compared to none in the honey group.	Weight:the honey-treated patients showed either static or positive weight gain during radiotherapy(p<0.05). RT interruption: treatment of four patients (20%) was interrupted among controls compared to none in the honey arm.
Su, et al. 2004 (USA) [31]	Aloe Vera	Incidence of maximal mucositis: aloe vera group was lower (p = 0.39); Duration of Grade 2 or worse mucositis: aloe vera group was lower (p = 0.4).	Score in soreness level: aloe vera group was lower(p = 0.89).	QOL score: aloe vera group was higher (p = 0.11).	Patients in both arms reported good compliance. No patients reported adverse effects, and none withdrew from the study because of side effects.	patients in both arms had the same outcomes along a variety of other dimensions, including percentage of weight loss, use of narcotic analgesic medications, iv. hydration requirement, use of antibiotics for oral infections, prolonged RT breaks, and overall treatment time.
Naidu et al. 2005 (India) [44]	Mucotrol™ (MF 5232)	Score: MF 5232 group had a significant reduction (WHO: p = 0.007,RTOG:P = 0.031).	NR	NR	In MF5232 treatment group: two patients had a mild localb urning sensation in mouth during the oral gel wafer administration, and withdrew from the study.	NR
Wu et al. 2007 (China) [50]	Qingre Liyan Decoction	Incidence: Qingre Liyan Decoction group had lower incidence of mucositis and higher effect in preventing than control group (P<0.05).	NR	NR	No adverse reaction on liver function or kidney function was found in either group. Patients in Qingre Liyan Decoction group were in good condition with normal spirits and intake of food and drinks.	The EGF in saliva, and CD4 and CD8 in the blood of patients in the trial group were higher than those in the control group (P<0.05).
Motallebnejad et al. 2008 (Iran) [25]	pure honey from Thymus and Astragale	Grade: the honey group had a significant reduction in mucositis and higher changes (p = 0.000).	NR	NR	In the honey group, four patients (20%) refuse t0 treat or take medication. In the control group three patients temporarily refused to continue the treatment.	Weight:weight loss was measured lesser in honey group(p = 0.000).
Rashad et al. 2009 (Egypt) [27]	pure honey from the clover plant Trifolium alexandrenum.	Incidence: the honey group had lower incidence of severe mucositis (p = 0.05).	NR	NR	In control group, five patients interrupted the therapy because of radiation mucositis, compared with none in the honey group. The median discontinuation time was seven days (range, four to 10 days).	RT interruption: five control group patients’ (25%) therapy was interrupted as a consequence of radiation mucositis, compared with none in the treatment group. Candida colonization:the honey group was lower (p = 0.003). Aerobic pathogenic bacteria: the honey group was lower (p = 0.007).
Maddocks-Jennings et al. 2009 (New Zealand) [37]	Essential oils (a 1:1 mix of maunka and kanuka oils)	Time of onset: essential oil gargle group were observed to have a delayed onset of muscositis (P = 0.05).	Essential oil gargle group had a reduction on pain and oral symptoms relative to placebo (gargling with water) and the control (‘usual care’) groups.	NR	All patients reported experiencing a range of symptoms oral and appetite related symptoms and the symptom severity tended to be lower in the essential oil group for the domain of excess secretions/nausea and vomiting.	Weight: patients in the essential oil group were seen to have less weight loss.
You et al. 2009 (Taiwan) [41]	Indigowood root	Incidence of severe OM: Indigowood root (IR) group was lower and siginificantly reduced the severity of radiation mucositis (p = 0.01).	NR	NR	Patients’ resting days did not show a significant difference (p = 0.06).	Clinical subjective symptoms: the severity of anorexia (p = 0.002) and swallowing difficulty (p = 0.002) were lower in the IR group. Blood content:white blood cell counts was lower in the IR group. Serum cytokines: the serum IL-6 level was much lower in the Indigowood root group at the first, fifth, and seventh week.
Putwatana et al. 2009 (Thailand) [32]	glycerin payayor	Time of onset: the Payayor group showed significantly later (P<0.001); Grade: the Payayor group severity were less (P<0.001).	Score: pain score (P<0.001) were less in the payayor group.	NR	In the payayor group, 10% of the Patients temporarily stop postpone radiation because of radiation adverse effects. Tiredness and fever were given as reasons for postponing treatment in the payayor group. Patients were significantly higher satisfaction with the solution	Weight: the payayor group had a higher average body weight (p = 0.000). Xerostomia, the feeling of dry mouth, and taste alteration:they were less in the payayor group with no difference.
Jayachandran et al. 2012 (India) [26]	pure honey (Dabur honey)	Score: the honey group had a significant reduction in mucositis compared with 0.15% benzydamine hydrochloride, 0.9% normal saline groups (p< 0.001)). Time of onset: the honey group delayed the onset of grade 1,and reduced the severity of grade 4.	NR	NR	NR	NR
Pawar et al. 2013 (India) [46]	SAMITAL	Grade:SAMITAL® group had significant improvements from baseline in OM grade. Score reduction: SAMITAL group had a greater reduction in mean OM score (p<0.05).	Pain score:SAMITAL group had a significantly greater reduction (p<0.05).	NR	In SAMITAL group, five reported nausea and vomiting. In detail three experienced severe vomiting and two had moderate vomiting; In the placebo group, five patients reported adverse events including nausea (n = 3), diarrhoea (n = 1) and cough (n = 1) and withdrew from the study before day 14. No adverse events were deemed to be related to treatment.	NR
Hawley et al. 2014 (Canada) [28]	manuka honey	Score of RTOG score (≧ 3): the proportions were similar in both arms (p = 0.7726).	No statistically significant difference	No statistically significant difference	The dropout rate was similar in both groups. Dropouts were mostly due to nausea.Patients in the placebo group tended to have a longer duration on the study than those in the honey group (median, 31 vs. 22 days). The majority of contactable dropouts reported nausea and the strong taste to be the biggest issues with both products. Two patients in the honey group reported a burning sensation in the mouth after application, which was not reported from any of the placebo patients.	NR
Sahebjamee et al. 2015 (Iran) [30]	aloe vera	Grade:no statistically significant differences(p = 0.35); Grade changes over time: no statistically significant differences(p = 0.09). Time of onset:no statistically significant differences(p = 0.97); Time of duration: no statistically significant differences (p = 0.98).	NR	NR	Two patients (15.4%) in the Aloe vera group experienced nausea. No patient reported any major adverse effect or withdrew.	NR
Mansourian et al. 2015 (Iran) [19]	Curcuma Longa	Incidence: curcuma longa topical gel group was lower (p<0.0001).	NR	NR	NR	The mean size of oral lesions, oral erythema and burning mouth sensation in the intervention group was significantly lower than the control group (P<0.001).
Bolouri et al. 2015 (Iran) [38]	propolis	score: the mucositis score at the end of each week in the propolis group was significantly lower. Grade: the mean rank at the end of each week in propolis group had significantly (P < 0.05) lower grade in all of the follow-ups.	NR	NR	NR	Weight: patients in proploils group had much more weight loss(p = 0.029), but xerostomia is not significantly different in two groups (P > 0.05).
Elyasi et al. 2016 (Iran) [35]	silymarin	Score:silymarin group had lower median scores of mucositis in at the end of the first to sixth week (p < 0.05). Time of onset:the silymarin group had a delay for mucositis development and progression.	NR	NR	Silymarin and placebo tablets were well tolerated,and no adverse effects relevant to silymarin administration was reported by the patients.	NR
Luo et al 2016 (China) [43]	Kangfuxin Solution	Incidence and grade:Kangfuxin Solution group were significantly reduce the incidence and severity of oral mucositd (p = 0.01) compared with the control group. Time of onset: Kangfuxin Solution group had longer time to different grade of oral mucositis occurrence (grade 1, 2, or 3) (p<0.01).	Kangfuxin Solution group showed lower incidence of oral pain than the control group (p< 0.01).	NR	No significant adverse events were observed.	Kangfuxin Solution group showed lower incidence of gastrointestinal mucositis than the control group (p< 0.01).
Marucci et al. 2017 (Italy) [47]	Faringel (propolis, aloe vera, calendula, and chamomile)	Incidence:Patients developed peak grade 3 mucositis with no difference between two groups (P = 0.665).	No statistically significant difference (p = 0.86).	NR	Seven patients (two patients receiving placebo; and five patients receiving Faringel) discontinued mouthwashes before treatment completion. Four patients (one patients receiving placebo; and three patients Faringel) complained of its taste or consistency. In three patients, the cause was not reported.	NR
Doğan et al. 2017 (Turkey) [34]	black mulberry molasses	Incidence and severity: the incidence and severity of oral mucositis were lower in the black mulberry molasses group (p < 0.05).	Average pain score was lower in the black mulberry molasses group. (p = 0.005).	Quality of life was higherr in the black mulberry molasses group(p < 0.00).	NR	NR
Aghamohammadi et al. 2018 (Iran) [39]	zataria	Score:the zataria group significantly decreased intensity (p<0.05). Incidence: the zataria group had a two fold decrease in the incidence of grades 3–4 OM. The use of the zataria affected the incidence of grades 3–4 OM to a relative risk ratio of 0.432.	Pain score in placebo group (P = 0.000) and zataria group (P = 0.003) were significantly different in weeks of treatment. Patients in the zataria group had a lower pain score at weeks 2 to 6 of treatmen	NR	NR	Weight loss (p = 0.000) and the need for analgesics (p = 0.010) and antibiotics (p = 0.037) were significantly lower in the zataria group.
Zheng et al. 2018 (China) [48]	Shuanghuabaihe Tablets	Incidence: Shuanghuabaihe tablets group had lower incidence (P = 0.0028). Time of onset: Median latency was 28 days in the 25 in Shuanghuabaihe group and 14 days in the placebo group (P<0.0001). Score: Compared with the placebo, Shuanghu:abaihe tablets significantly reduced oral mucositis severity scores (P<0.0001), full-time nurses (P<0.0001) and patients (soreness of the mouth and throat: P<0.0001).	Compared with the placebo, Shuanghu:abaihe tablets significantly reduced soreness of the mouth and throat (P<0.0001).	NR	Mild/moderate gastrointestinal reactions (such as gasteremphraxis, nausea, vomiting and gastrointestinal tract responses) were associated with the Shuanghuabaihe tablets, with an overall incidence of 3.33%. And no serious adverse events associated with Shuanghuabaihe tablets were observed.	Shuanghuabaihe tablets significantly reduced full-time nurses (P<0.0001).
Charalambous et al. 2018 (Cyprus) [29]	thyme honey	Grade and severity: the honey group were graded lower (p < 0.001). The upward trend in the severe OM prevalence in the control group and the downward trend in the intervention group throughout the 7-week period.	Pain reduction were much more in honey group than in normal saline group.(Likert scale)	The Overall Health and QoL in the Intervention group increased at 1st month after the completion of radiotherapy and at 6 months.	Three patients from the honey group and four patients from the control group died during the treatment period. Four patients from the honey group discontinued the intervention as a result of secondary complications.	Weight: Both groups had a decrease in their weight over the 7-week period, nevertheless patients in the normal saline group lost more weight compared to those in honey group.
Wang et al. 2018 (China) [49]	CHIN	Grade: CHIN group had prominent remission of grade on each observing point compared with those in control group (P <0.01).	CHIN group had prominent remission of oral pain(P <0.01).	NR	UR	Xerostomia: Xerostomia was decreased notably in treatment group compared with control group (P < .01). Body mass index: Body mass index in CHIN group exhibited advantage over control group after radiotherapy, but there was no statistical significance(p>0.05).
Delavarian et al. 2019 (Iran) [21]	curcumin	Grade: the curcumin group was lower (P < 0.05); Mean severity: the curcumin group was lower(p = 0.005); Time of onset: the curcumin group had a delay in the onset of OM (P = 0.002).	NR	NR	The administration of nanocurcumin did not cause detectable side effects or discomfort. One patient in curcumin group and 2 patients in the placebo group refused to continue their radiotherapy treatment.	Weight: the curcumin group had a lower average loss of weight (P = 0.003).
Arun et al. 2020 (India) [23]	curcumin	Grade: the curcumin group was higher from the third week onwards (p < 0.001).	NR	NR	NR	NR
Shah et al. 2020 (India) [20]	curcumin	Risk of the onset: the curcumin group was lower instantaneous risk of getting the onset of RIOM (hazard ratio 0.5); Time of onset: the curcumin group showed a significant delay of onset of RIOM;	NR	NR	One patient in each study groups reported burning sensation as side effect after 3 weeks of mouthwash usage.	NR
Mamgain et al. 2020 (India) [52]	conventional treatment +Glycyrrhiza glabra(Yashtimadhu)	Incidence: the yashtimadhu group was lower at each accessment time point. Time of onset: The onset of Grade 1 mucositis was early in conventional group (mean = 15th day) as compared to yashtimadhu and honey groups.	NR	NR	Unplanned treatment breaks and hospitalization of patients were reduced with the use of yashtimadhu as compared to other two groups.	NR
Soltani et al. 2020 (Iran) [42]	plantago major L	Grade:the Plantago major L group had lower severity of mucositis (p< 0.05).	The Plantago major L group had less pain scores (p< 0.05).	NR	In the intervention group, one patient had transient nausea and one suffered mild abdominal cramp, One patient in the placebo group developed tolerable headache, but all of them continued their intervention.	NR
Arantes et al. 2021 (Brazil) [24]	FITOPROT	Severity: FITOPROT group presented a gradual decrease from the 15th to the 21st RT sessions (p = 0.03, WHO; p = 0.02,NCI).	NR	similar reductions	No patient reported adverse effects.	Nitrite level and pro-inflammatory cytokines: in salivary nitrite concentration at the 15th RT session when compared to their initial concentration, whereas 60.0% (n = 9) of the placebo group showed an increase in this inflammatory mediator (p = 0.04). In salivary levels of IL-8 intervention group, although no statistical significancEwas reached, there was a reduction,between the 15th and 21st RT sessions (p > 0.05)
Hosseini et al. 2021 (Iran) [36]	silymarin	Score: The median scores were not significantly different between groups at the end of the sixth week (p > 0.05). The scores increased significantly in both groups during radiotherapy. Risk:silymarin may decelerate its progression especially after 4 weeks of use.	NR	NR	Most of the patients in silymarin group complained its unpleasant taste (80%), four patients experienced nausea and one patient abdominal pain.	NR
Ebert et al. 2021 (Germany) [40]	Cystus® tea	Time of onset: There was no significant difference between the two groups in latency (p = 0.75) and frequency (p = 0.85) of the occurrence of mucositis grade 3. Incidence: No statistically significant differences were found between groups, with a low incidence of mucositis grade 3.	NR	NR	Two did not complete the course of radiotherapy (because of noncompliance due to alcohol-induced delirium and unexpected death during therapy).	Occurrence of dental pathologies appeared to increase over time after radiotherapy.
Soni et al. 2022 (India) [22]	turmeric extract	Incidence: BTF group was significantly lower in the incidence of grade 3 toxicity of oral mucositis, than placebo group (BTF 1 g/day (p = 0.011), BTF1.5 g/day (p = 0.004))	Grade of pain: In arms BTF 1g/day and BTF 1.5g/day,patients had a lower grade and had significant lower incidence and severity of pain. copared to placebo arm.	NR	Eighteen of 60 patients (30%) had significant treatment interruption/gap, Fifteen % of patients in arm BTF 1g/day had significant treatment interruption in radiotherapy compared to 20% of patients in arm BTF 1.5g/day (p = 0.677) and 55% of patients in placebo group.	In arms BTF 1g/day and BTF 1.5g/day,patients had a lower grade and had significant reduction of dysphagia and dermatitis copared to placebo arm. Requirement of tube feeding was be decreased in arms BTF 1g/day (25%) and BTF 1.5g/day (20%) compared to placebo arm (60%). Weight: More patients (75% patients) in arm placebo developed significant weight loss (>3 kg from baseline) during the treatment than patients in arm A (60% of patients) (p = 0.025) and arm B (25% of patients) (p = 0.002).
Fasanaro et al. 2022 (Italy) [45]	SAMITAL	Incidence: the SAMITA group was lower (p = 0.0136); Time of onset: no statistically significant difference (p = 0.2429).	The use of non-steroidal anti-inflammatory drugs or opioids to relieve oral mucositis pain was not different between the two arms (overall p = 0.2135)	Patients in SAITAL group had a better quality of life.	Five patients (three in the SAMITAL group and two in the placebo group) had a definitive radiotherapy interruption; The compliance with SAMITA was lower with no statistical difference.	Weight: the SAMITAL group had a significantly decreased starting from week 3 (p = 0.0118) and from week 1(p = 0.004).
Sari et al. 2022 (Turkey) [33]	arm A: grape molasses gargle arm B: black mulberry olasses	Score:similar among the groups.	similar	Global health score was higher in black mulberry molasses group at the 6th week of RT compared to that of grape molasses group. There was no significant difference for any of the EORTC H&N35 module.	NR	Weight: a significant decrease in the mean body weight was observed in patients in all groups. Both grape molasses group and black mulberry molasses group improved outcomes for swallowing, opening mouth, and weight loss without significant difference.
Martins et al. 2023 (Brazil) [18]	FITOPROT+PBM+POCP	Incidence: the FITOPROT group had higher incidence of severe om.	NR	QOL score: FOTOPROT had a higher variation without statistical significance.	A greater adherence to the proposed medications was observed at the 21st RT session in group using of FITOPROT. All patients who developed OM were able to use the phytomedication without diluting it, and they reported a sensation of refreshment during its use.	RT interruption: similar.
Shirazian et al. 2023 (Iran) [17]	green tea	Grade:the green tea group had significant reduction of the severity of mucositis during weeks 5–8 (p<0.001).	similar	NR	NR	NR

Of the included studies, 7 studies reported Curcuma longa [18–24], 6 studies reported honey [16, 25–29] (including pure honey from tea plant Camellia sinensis [16], pure honey from Thymus and Astragale [25], pure honey (Dabur honey) [26], pure honey from the clover plant Trifoliumalexandrenum [27], manuka honey [28], and thyme honey [29]), 3 studies reported aloe vera [30–32], 2 studies reported black mulberry molasses [33, 34], and 2 studies reported FITOPROT [18, 24] (an adhesive herbal remedy that contains glycerinated extract of Bidens pilosa L. and curcuminoids from Curcuma longa L. [18]), 2 studies reported Silymarin [35, 36], and 1 study reported essential oils of manuka and kanuka [37]. Seven studies reported on propolis [38], Zataria [39], green tea [17], Cystus® tea [40], Indigowood root [41], Plantago major L [42], and Kangfuxin solution [43]. The remaining studies reported herbal medicines in treating oral mucositis: Mucotrol™ [44] (a herbal remedy made up of centella asiatica, sorbitol, magnesium stearate, acesulfame K, aloe vera sp., and glycyrrhizin extract [44]), SAMITAL® [45, 46] (a blend of three botanical medicine extracts from the roots of Echinacea angustifolia and V accinium myrtillus (bilberry, and the fruits of Macleaya cordata [46]), Faringelb (comprising propolis, Aloe vera, calendula, chamomile, and honey) [47], Shuanghuabaihe tablets (extracts from a range of Chinese herbs, such as Isatidis radix, Asari radix etrhizoma, serpentine bile, Rehmanniae radix, Liliibulbus, Arnebiae radix, Rhizoma Coptidis, and Lonicerae Japonicae Flos) [48], CHIN (Chining decoction modified from Liangge San consisting of Radix Liriopes, Radix Scrophulariae, Lumbricus, Scutellaria, Rhubarb, Licorice, Mint, and Forsythia, Red peony root) [49], Qingre Liyan decoction (which includes the Astragalus, Crocus sativus, Ophiopogon flexuosus, Panax ginseng, Draba hebecarpa, Ligustrum, Radix et Rhizoma Glycyrrhizae, Radix Angelicae Sinensis and Glycyrrhizae) [50].

### Oral mucositis measurement

Assessment of oral mucositis was performed using scales for assessing the degree of mucositis. The scales used for assessment included the World Health Organization (WHO) scale [17, 21, 23, 26–28, 32, 35, 42, 44, 45, 50, 51], the Common Terminology Criteria scale (NCI-CTC) [16, 21–23, 33–36, 38, 41, 43, 47–49], the Radiation Therapy Oncology Group (RTOG) [16, 28, 29, 31, 33, 37, 40, 44, 50, 52] scale (used for the Cooperative Group Common Toxicity Criteria), the Oral Mucositis Assessment Scale (OMAS) [34, 45, 48] and the Oral Assessment Guide (OAG) [25, 28, 39, 45].

All trials reported the efficacy of natural products for treating oral mucositis caused by radiation therapy. Twenty-six trials reported a reduction in mucositis grade and scale score and a decrease in the degree of mucositis. Sixteen studies (including Aloe Vera [31], Curcuma longa [19, 22, 23], FITOPROT [18], Honey [27], Yashtimadhu [52], black mulberry [34], SAMITAL® [45], Indigowood Root [41], Faringel [47], Zataria [39], Shuaghuabaihe Tablets [48], Cycus tea [40], QingReliyanb decoction [50], and Kangfuxin solution [43]) revealed a possible reduction in the incidence of mucositis during radiotherapy. Thirteen studies, including those involving Aloe vera (30,32), Curcuma longa [19–21], Honey (Dabur honey) [26], Glycyrrhiza glabra (Yashtimadhu) [52], SAMITAL® [45], essential oils [37], silymarin [35], Shuaghuabaihe tablets [48], Cycus tea [40], and Kangfuxin [43], reported that using the natural products postponed the beginning of oral mucositis, regarding which the Cycus tea and SMITAL studies found no discernible differences. Three trials reported that turmeric [20], zataria [39], and silymarin [36] reduced the risk of mucositis onset, and there was one report of aloe vera shortening the duration of mucositis with no discernible significant difference.

### Pain reduction

Regarding the outcome of pain (mainly assessed by the visual analog scale (VAS)), eighteen trials reported results. Thirteen of these studies showed a significant reduction, including curcumin [22], FITOPROT [24], thyme honey [29], black mulberry molasses [34], SAMITAL® [45, 46], essential oils [37], Payayor [32], Zataria [39], Shuanghuabaihe tablets [48], Plantago major L. [42], CHIN [49] and Kangfuxin solution [43]. Five studies [17, 28, 31, 33, 47] showed no significant effect.

### Quality of life assessment

A total of seven trials assessed patients’ quality of life, mainly using the UW-QOL, EORTC QLC-C30 and EORTC QLC-H&N35 scales. Two [28, 31] of the trials of aloe vera and manuka honey showed no significant results in terms of pain reduction, while the other six [18, 24, 29, 34, 45, 46] trials of aloe vera, honey, black mulberry molasses, FITOPROT and SAMITAL® showed a significant reduction in pain symptoms.

### Other symptoms

When assessing the effects on other symptoms, nine trials revealed that curcumin (19, 22), honey [16, 25, 29], black mulberry molasses [33], Payayor [32], propolis [38] and zataria [39] were able to reduce weight loss, although these effects were not significant in studies on payayor [32] or propolis [38]. Four studies revealed that SAMITAL® [45], payayor [32], propolis [38], and the herbal compound CHIN [49] were effective at reducing xerostomia. Four studies revealed that curcumin [22], Indigowood root [41], SAMITAL® [45], and black mulberry molasses [33] reduced the symptoms of dysphagia. FITOPROT [18], honey [16, 27], and SAMITAL® [45] were useful for decreasing the interruptions of radiotherapy. In addition, patients using Indigowood root were found to have significantly lower levels of leukocytes in the blood and IL-6 in the serum [41], and FITOPROT reduced salivary nitrite and IL-8 levels [24]. Honey significantly reduced the number of aerobic pathogenic bacteria as well as the extent of Candida colonization [27].

### Acceptability

Twenty-five trials reported acceptability. In the studies by Su [31], Soni [22], Fasanaro [45] et al., good compliance was reported for patients treated with aloe vera, curcumin, and SAMITAL®. In the studies by Elyasi [35], Soltani [42] et al., patients showed good tolerance to silymarin and Plantago major L. In a study of Manuka honey, some subjects withdrew from the study due to the strong taste and moderate to severe nausea [28].

### Adverse events

Among the trials that reported adverse events, nausea and vomiting were reported in the trials of aloe vera and Manuka honey [28, 30–32], and nausea, burning sensation in the mouth and gastrointestinal reactions were reported in the trials of SAMITAL®, silymarin, Plantago major L. and Shuanghuabaihe tablets [35, 36, 42, 45, 46, 48]. For Plantago major L., the adverse effects reported were gastrointestinal reactions and burning sensations in the mouth [42]. The gastrointestinal reactions were mainly diarrhea with mild abdominal pain and cramps. Importantly, it is difficult to distinguish whether these side effects are caused by natural medicines or cancer radiotherapy, and it is necessary to report side effects consistently in each trial so that researchers can accurately assess treatment-related toxicity to minimize bias.

### Risk of bias

**Fig 2:**
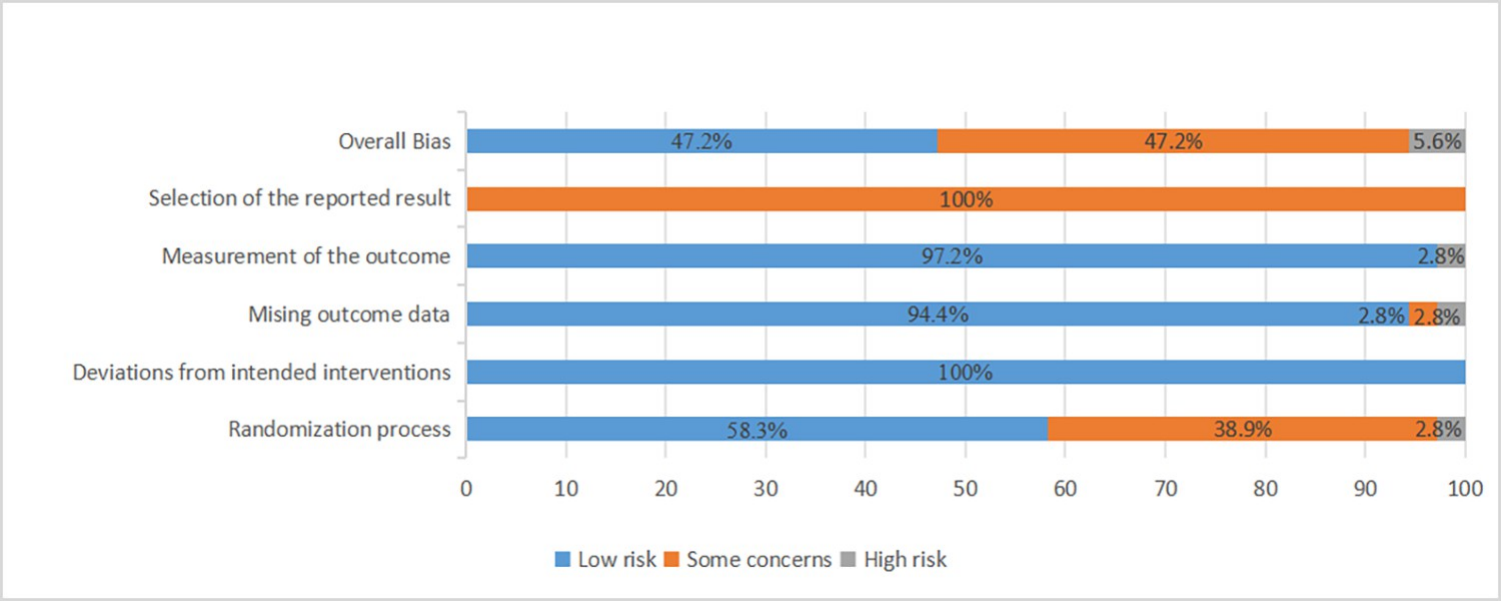
Summary of the risk of bias.

**Fig 3:**
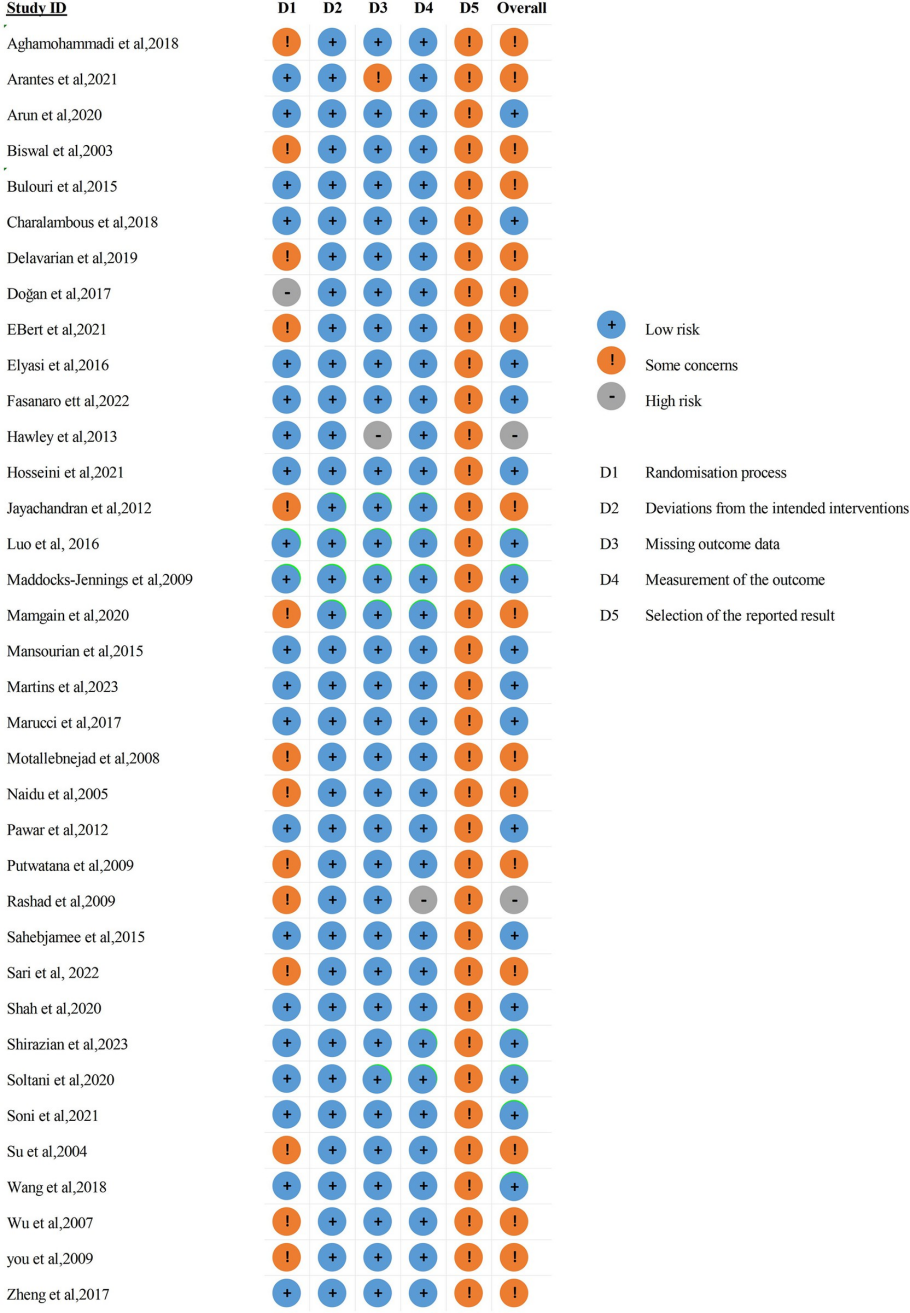
Assessment of the risk of bias in clinical trials.

The methodological quality of the 36 included studies is illustrated in **Fig 2** and assessment of the risk of bias in each trial is showed in **Fig 3**. The revised risk-of-bias tool ROB 2 was used to evaluate the risk of bias in each included trial. In domain 5, every study received a rating of "some concern." It is unknown whether articles of clinical trials or even records were analyzed in accordance with a predetermined plan that was finished before information on nonhidden outcomes was available for examination.

## Discussion

The present study investigated the role of natural products in radiotherapy-induced oral mucositis. Natural products can simultaneously target different primary pathways that support pathobiology and affect the pathogenesis of mucositis at different levels without compromising the efficacy of antitumor regimens [53, 54]. In this respect, natural products are very good alternative therapies compared to synthetic drugs.

In this article, we categorized and reported the results of the effects of natural products on relieving oral mucositis, reducing pain, improving quality of life and ameliorating comorbidities such as xerostomia and dysphagia. Studies have shown that turmeric, honey, Zataria, Indigowood root, black mulberry molasses, SAMITAL®, Kangfuxin solution, and the Chinese herbal compounds Shuanghuabaije tablets, CHIN, and Qingreliyan decoction have favorable ameliorative therapeutic effects on radiotherapy-induced oral mucositis. However, most studies’ outcomes were frequently constrained by the small number of participants involved and/or lack of standardized intervention methodology. Even comparisons between the same drugs are difficult because of the limitations of the different concentrations used, dosages used, and extraction methods, such as essential oils (from manuka and kanuka), propolis, aloe vera, bluebell root and silymarin. The essential oils appeared to be effective in preventing OM due to their wound-healing, anti-inflammatory, antimitotic, analgesic, and antibacterial qualities, but an important limitation of the study was the small sample size [37]. Although the use of very small doses of Manuka and Kanuka in a gargle may have a favorable effect on the development of radiation-induced mucositis, additional trials are needed to corroborate these findings, according to the findings of feasibility studies. Propolis is rich in flavonoids, which have healing, antiulcer, anti-inflammatory and antioxidant effects, and the findings of this study support this hypothesis regarding the management of oral mucositis; however, the small sample size was also the primary drawback of this study, and further research is needed to clarify the potential radioprotective mechanisms involved [38, 55, 56]. Aloe vera has been shown to have anti-inflammatory, antimicrobial, antioxidant, antitumor, skin-protective and wound-healing pharmacological effects in several *in vitro* studies [57]; however, of the two studies included in this review, the study by Su et al. revealed no discernible impact of oral aloe vera solution in the treatment of mucositis [31]. A study by Sahebjamee et al. revealed that aloe vera delayed the onset of mucositis and severe mucositis [30]. There is still a need for extensive mechanistic studies on aloe vera extracts and further determination of the therapeutic effect of aloe vera by increasing the sample size, subdividing the type of primary site and conducting clinical trials with different routes of administration. Indigowood root has anti-inflammatory properties, and the component it contains, Indigofera, is thought to lessen the degree of dysphagia, anorexia, and oral mucositis caused by radiation therapy; however, the specific mechanism and pathway by which Indigowood root reduces radiotherapy-induced mucositis requires further analysis [41]. Silymarin is extracted from Milk thistle (Silybum marianum L.). The extract contains several flavonolignans, the major component being silymarin, which is associated with many pharmacological properties, including antioxidant, anti-inflammatory, immunomodulatory and hepatoprotective properties [58, 59]. The efficacy of these agents in preventing radiotherapy-induced mucositis was increased in the included studies, but clinical trials with larger sample sizes, especially with different doses, durations and nanoformulations of silymarin, are advised to verify this possible effect [35].

This study has certain limitations, and there is room for methodological improvement in RCTs investigating natural products. First, because not all protocols or registration information from the included trials were available, the articles may have publication bias due to unpublished unfavorable or negative results. Some of the trials did not describe how allocation was concealed, and the presence of some drugs, such as honey, which could not be blinded, made the literature less reliable. Second, the WHO, RTOG, OMAS, NCI-CTCAE and OAG oral mucositis grading scales were used, the grading criteria were not standardized, and only a few trials reported that grading was performed by a person with a professional background in mucositis, making it difficult to compare the staging and grading of the disease. In addition, safety was described in general terms and not specifically documented using the relevant scales. Only one of the trials included in the review described the effect of therapy in combination with other conventional therapies for the treatment of radiation-related oral mucositis, and safety studies of natural products combined with other conventional and/or naturopathic therapies are incomplete. The safety and efficacy of these treatments need to be investigated further.

In conclusion, according to our review of trials, the application of natural remedies in the treatment of radiotherapy-induced oral mucositis exhibited good efficacy and safety. The vast majority of these natural products were well accepted, but in the manuka honey trial, patients withdrew from the study due to nausea and unpleasant taste. Nonetheless, there is a need to extend investigations by increasing the sample size, improving the dosing regimen, and assessing compliance with the CONSORT guidelines for the herbs and Chinese herbal medicines included, thereby enhancing the quality of the clinical trials.

## Conclusion

The results of the present systematic review reveal that curcumin, honey, zataria, Indigowood root, black mulberry molasses, aloe vera, silymarin, Kangfuxin solution and the herb compounds Mucotrol™ FITOPROT, SAMITAL®, Shuanghuabaihe tablets, Qingreliyan decoction, and CHIN have favorable effects on the treatment of radiotherapy-induced oral mucositis. Additionally, we support the use of natural products to treat radiotherapy-induced oral mucositis. However, there is still a need for additional studies focusing on the safety of these traditional medicines and their efficacy and safety when used in combination with other conventional and/or naturopathic therapies.

## Supporting information

S1 Checklist PRISMA 2020 checklist. (DOCX)

S1 File Search strategy. (DOCX)
