# Supplementary material for: Clinical efficacy and applicability of natural products in the treatment and prevention of radiotherapy-induced oral mucositis: A systematic review
Source: PLoS One. 2024 May 23;19(5):e0303988. doi: 10.1371/journal.pone.0303988 (PMC11115216; doi:10.1371/journal.pone.0303988)
Supplement: S1 File — (DOCX) [file pone.0303988.s002.docx]

Search strategy

The main terms and detailed search strategies used for each database are as follows.

**the Cochrane Library (n=83) :**

| #1 | MeSH descriptor: [Stomatitis] explode all trees |
| --- | --- |
| #2 | (Stomatitis):ti,ab,kw OR (Stomatitides):ti,ab,kw OR (Oral Mucositis):ti,ab,kw OR (Mucositides, Oral):ti,ab,kw OR (Oral Mucositides):ti,ab,kw OR (Oromucositis):ti,ab,kw OR (Oromucositides):ti,ab,kw OR (Mucositis, Oral):ti,ab,kw |
| #3 | MeSH descriptor: [Radiotherapy] explode all trees |
| #4 | (radiotherapy):ti,ab,kw OR (Radiation Treatment):ti,ab,kw OR (Therapy, Radiation):ti,ab,kw OR (Treatment, Radiation):ti,ab,kw OR (Therapies, Radiation):ti,ab,kw OR (Radiation Therapies):ti,ab,kw OR (Radiation Treatments):ti,ab,kw OR (Radiation Therapy):ti,ab,kw OR (Radiotherapies):ti,ab,kw OR (Targeted Radiation Therapy):ti,ab,kw OR (Targeted Radiotherapies):ti,ab,kw OR (Radiation Therapies, Targeted):ti,ab,kw OR (Targeted Radiation Therapies):ti,ab,kw OR (Radiotherapy, Targeted):ti,ab,kw OR (Radiotherapies, Targeted):ti,ab,kw OR (Radiation Therapy, Targeted):ti,ab,kw OR (Therapies, Targeted Radiation):ti,ab,kw OR (Targeted Radiotherapy):ti,ab,kw OR (Therapy, Targeted Radiation):ti,ab,kw |
| #5 | MeSH descriptor: [Biological Products] explode all trees |
| #6 | (Biological products):ti,ab,kw OR (Biologic Drug):ti,ab,kw OR (Drugs, Biological):ti,ab,kw OR (Biologic Medicines):ti,ab,kw OR (Pharmaceuticals, Biologic):ti,ab,kw OR (Biologicals):ti,ab,kw OR (Biologic Pharmaceuticals):ti,ab,kw OR (Biological Medicines):ti,ab,kw OR (Biologic Drugs; Biopharmaceuticals):ti,ab,kw OR (Drug, Biologic):ti,ab,kw OR (Biological Drugs):ti,ab,kw OR (Biopharmaceutical):ti,ab,kw OR (Biological Drug):ti,ab,kw OR (Drugs, Biologic):ti,ab,kw OR (Medicines, Biologic):ti,ab,kw OR (Medicines, Biologic):ti,ab,kw OR (Biologic):ti,ab,kw OR (Medicines, Biological):ti,ab,kw OR (Medicine, Biological):ti,ab,kw OR (Biological):ti,ab,kw OR (Biologics):ti,ab,kw OR (Drug, Biological):ti,ab,kw OR (Biological Medicine):ti,ab,kw OR (Product, Natural):ti,ab,kw OR (Natural Product):ti,ab,kw OR ( Natural Products):ti,ab,kw OR (Products, Biological):ti,ab,kw OR (Biologic Product):ti,ab,kw OR (Biological Product):ti,ab,kw OR (Product, Biological):ti,ab,kw OR (Biologic Products):ti,ab,kw OR (Product, Biologic):ti,ab,kw OR (Natural Compounds):ti,ab,kw OR (Natural Compound):ti,ab,kw |
| #7 | MeSH descriptor: [Phytotherapy] explode all trees |
| #8 | (Herbal Medicine):ti,ab,kw OR (Medicine, Herbal):ti,ab,kw OR (Herbalism):ti,ab,kw OR (Traditional Medicine Practitioners):ti,ab,kw OR (Naturopathy):ti,ab,kw OR (Plant Extracts):ti,ab,kw OR (Plants, Medicinal):ti,ab,kw OR (Herbal Therapy):ti,ab,kw OR (Herb Therapy):ti,ab,kw OR (Drugs, Chinese Herbal):ti,ab,kw OR (Pharmacognosy):ti,ab,kw OR (Plant Extracts):ti,ab,kw OR (Ethnobotany):ti,ab,kw OR (Ethnopharmacology):ti,ab,kw OR (Flower Essences):ti,ab,kw |
| #9 | #1 OR #2 |
| #10 | #3 OR #4 |
| #11 | #5 OR #6 OR #7 OR #8 |
| #12 | #9 AND #10 AND #11 |

**Web of Science (n=330) :**

| #1 | stomatitis OR stomatitides OR oral mucositis OR mucositides, oral OR oral mucositides OR oromucositis OR oromucositides OR mucositis, oral |
| --- | --- |
| #2 | TS=(radiotherapy or Radiation Treatment or Therapy, Radiation or Treatment, Radiation or Therapies, Radiation or Radiation Therapies or Radiation Treatments or Radiation Therapy or Radiotherapies or Targeted Radiation Therapy or Targeted Radiotherapies or Radiation Therapies, Targeted or Targeted Radiation Therapies or Radiotherapy, Targeted or Radiotherapies, Targeted or Radiation Therapy, Targeted or Therapies, Targeted Radiation or Targeted Radiotherapy or Therapy, Targeted Radiation) |
| #3 | TS=(Herbal Medicine or Medicine, Herbal or Herbalism or Traditional Medicine Practitioners or Phytotherapy or Naturopathy or Plant Extracts or Plants, Medicinal or Herbal Therapy or Herb Therapy or Drugs, Chinese Herbal or Pharmacognosy or Plant Extracts or Ethnobotany or Ethnopharmacology or Flower Essences or natural product or Herbal Medicine or Medicine, Herbal or Herbalism or Traditional Medicine Practitioners or Phytotherapy or Naturopathy or Plant Extracts or Plants, Medicinal or Herbal Therapy or Herb Therapy or Drugs, Chinese Herbal or Pharmacognosy or Plant Extracts or Ethnobotany or Ethnopharmacology or Flower Essences or natural product or natural products or natural agent or natural agents or natural compound or natural compounds or biologocal product or biologocal products |
| #4 | #1 AND #2 AND #3 |

**PubMed (n=141) :**

| #1 | ((((((stomatitis[Title/Abstract]) OR (stomatitides[Title/Abstract])) OR (oral mucositis[Title/Abstract])) OR (mucositides oral[Title/Abstract])) OR (oral mucositides[Title/Abstract])) OR (oromucositis[Title/Abstract])) OR (mucositis, oral[Title/Abstract]) |
| --- | --- |
| #2 | ((((((((((((((((((radiotherapy[Title/Abstract]) OR (radiation treatment[Title/Abstract])) OR (therapy, radiation[Title/Abstract])) OR (treatment, radiation[Title/Abstract])) OR (therapies, radiation[Title/Abstract])) OR (radiation therapies[Title/Abstract])) OR (radiation treatments[Title/Abstract])) OR (radiation therapy[Title/Abstract])) OR (radiotherapies[Title/Abstract])) OR (targeted radiation therapy[Title/Abstract])) OR (targeted radiotherapies[Title/Abstract])) OR (radiation therapies[Title/Abstract])) OR (targeted radiation therapies[Title/Abstract])) OR (radiotherapy, targeted[Title/Abstract])) OR (radiotherapies, targeted[Title/Abstract])) OR (radiation therapy, targeted[Title/Abstract])) OR (herapies, targeted radiation[Title/Abstract])) OR (targeted radiotherapy[Title/Abstract])) OR (therapy, targeted radiation[Title/Abstract]) |
| #3 | (((((((((((((((((((((((Herbal Medicine[Title/Abstract]) OR (Medicine, Herbal[Title/Abstract])) OR (Herbalism[Title/Abstract])) OR (Traditional Medicine Practitioners[Title/Abstract])) OR (Phytotherapy[Title/Abstract])) OR (Naturopathy[Title/Abstract])) OR (Plant Extracts[Title/Abstract])) OR (Plants, Medicinal[Title/Abstract])) OR (Herbal Therapy[Title/Abstract])) OR (Herb Therapy[Title/Abstract])) OR (Traditional Medicine Practitioners[Title/Abstract])) OR (Drugs, Chinese Herbal[Title/Abstract])) OR (Pharmacognosy[Title/Abstract])) OR (Plant Extracts[Title/Abstract])) OR (Plants, Medicinal[Title/Abstract])) OR (Ethnobotany[Title/Abstract])) OR (Ethnopharmacology[Title/Abstract])) OR (Flower Essences[Title/Abstract])) OR (natural product[Title/Abstract])) OR (natural products[Title/Abstract])) OR (natural agent[Title/Abstract])) OR (natural agents[Title/Abstract])) OR (natural compound[Title/Abstract])) OR (natural compounds[Title/Abstract]) |
| #4 | ("1900/01/01"[Date - Publication] : "3000"[Date - Publication]) |
| #5 | (((((((((stomatitis[Title/Abstract]) OR (stomatitides[Title/Abstract])) OR (oral mucositis[Title/Abstract])) OR (mucositides oral[Title/Abstract])) OR (oral mucositides[Title/Abstract])) OR (oromucositis[Title/Abstract])) OR (mucositis, oral[Title/Abstract])) AND (((((((((((((((((((radiotherapy[Title/Abstract]) OR (radiation treatment[Title/Abstract])) OR (therapy, radiation[Title/Abstract])) OR (treatment, radiation[Title/Abstract])) OR (therapies, radiation[Title/Abstract])) OR (radiation therapies[Title/Abstract])) OR (radiation treatments[Title/Abstract])) OR (radiation therapy[Title/Abstract])) OR (radiotherapies[Title/Abstract])) OR (targeted radiation therapy[Title/Abstract])) OR (targeted radiotherapies[Title/Abstract])) OR (radiation therapies[Title/Abstract])) OR (targeted radiation therapies[Title/Abstract])) OR (radiotherapy, targeted[Title/Abstract])) OR (radiotherapies, targeted[Title/Abstract])) OR (radiation therapy, targeted[Title/Abstract])) OR (herapies, targeted radiation[Title/Abstract])) OR (targeted radiotherapy[Title/Abstract])) OR (therapy, targeted radiation[Title/Abstract]))) AND ((((((((((((((((((((((((Herbal Medicine[Title/Abstract]) OR (Medicine, Herbal[Title/Abstract])) OR (Herbalism[Title/Abstract])) OR (Traditional Medicine Practitioners[Title/Abstract])) OR (Phytotherapy[Title/Abstract])) OR (Naturopathy[Title/Abstract])) OR (Plant Extracts[Title/Abstract])) OR (Plants, Medicinal[Title/Abstract])) OR (Herbal Therapy[Title/Abstract])) OR (Herb Therapy[Title/Abstract])) OR (Traditional Medicine Practitioners[Title/Abstract])) OR (Drugs, Chinese Herbal[Title/Abstract])) OR (Pharmacognosy[Title/Abstract])) OR (Plant Extracts[Title/Abstract])) OR (Plants, Medicinal[Title/Abstract])) OR (Ethnobotany[Title/Abstract])) OR (Ethnopharmacology[Title/Abstract])) OR (Flower Essences[Title/Abstract])) OR (natural product[Title/Abstract])) OR (natural products[Title/Abstract])) OR (natural agent[Title/Abstract])) OR (natural agents[Title/Abstract])) OR (natural compound[Title/Abstract])) OR (natural compounds[Title/Abstract]))) AND (("1900/01/01"[Date - Publication] : "3000"[Date - Publication])) |

**Embase (n=100) :**

| #1 | 'stomatitis'/exp |
| --- | --- |
| #2 | 'oral mucositis'/exp |
| #3 | 'stomatitides':ab,ti OR 'mucositides, oral':ab,ti OR 'oral mucositides':ab,ti OR 'oromucositis':ab,ti OR 'oromucositides':ab,ti OR  'mucositis, oral':ab,ti |
| #4 | 'radiotherapy'/exp OR 'radiotherapy' |
| #5 | 'radiotherapy':ab,ti OR 'radiation treatment':ab,ti OR 'therapy, radiation':ab,ti OR 'treatment, radiation':ab,ti OR 'therapies, radiation':ab,ti OR 'radiation therapies':ab,ti OR 'radiation treatments':ab,ti OR 'radiation therapy':ab,ti OR 'radiotherapies':ab,ti OR 'targeted radiation therapy':ab,ti OR 'targeted radiotherapies':ab,ti OR 'radiation therapies,targeted':ab,ti OR 'targeted radiation therapies':ab,ti OR 'radiotherapy, targeted':ab,ti OR 'radiotherapies,targeted':ab,ti OR 'radiation therapy,targeted':ab,ti OR 'therapies, targeted radiation':ab,ti OR 'targeted radiotherapy':ab,ti OR 'therapy, targeted radiation':ab,ti |
| #6 | 'herbal medicine'/exp OR 'herbal medicine' |
| #7 | 'herbaceous agent'/exp |
| #8 | 'phytotherapy'/exp OR 'phytotherapy' |
| #9 | 'natural product'/exp |
| #10 | 'medicine, herbal':ab,ti OR herbalism:ab,ti |
| #11 | 'naturopathy':ab,ti OR 'herbal therapy':ab,ti OR 'herb therapy':ab,ti OR 'drugs, chinese herbal':ab,ti OR pharmacognosy:ab,ti OR 'plant extracts':ab,ti OR 'plants, medicinal':ab,ti OR ethnobotany:ab,ti OR ethnopharmacology:ab,ti OR 'flower essences':ab,ti |
| #12 | 'natural products':ab,ti OR 'natural agent':ab,ti OR 'natural agents':ab,ti OR 'natural compound':ab,ti OR 'natural compounds':ab,ti |
| #13 | #1 OR #2 OR #3 |
| #14 | #4 OR #5 |
| #15 | #6 OR #7 OR #8 OR #9 OR #10 OR #11 OR #12 |
| #16 | #13 AND #14 AND #15 |
| #17 | [<1966-2023]/py |
| #18 | #16 AND #17 |

**OVID (n=30) ：**

| 1 | (stomatitis or stomatitides or oral mucositis or mucositides, oral or oral mucositides or oromucositis or oromucositides or mucositis, oral).ti,ab,kw. |
| --- | --- |
| 2 | (radiotherapy or Radiation Treatment or Therapy, Radiation or Treatment, Radiation or Therapies, Radiation or Radiation Therapies or Radiation Treatments or Radiation Therapy or Radiotherapies or Targeted Radiation Therapy or Targeted Radiotherapies or Radiation Therapies, Targeted or Targeted Radiation Therapies or Radiotherapy, Targeted or Radiotherapies, Targeted or Radiation Therapy, Targeted or Therapies, Targeted Radiation or Targeted Radiotherapy or Therapy, Targeted Radiation).ti,ab. |
| 3 | (radiotherapy or Radiation Treatment or Therapy, Radiation or Treatment, Radiation or Therapies, Radiation or Radiation Therapies or Radiation Treatments or Radiation Therapy or Radiotherapies or Targeted Radiation Therapy or Targeted Radiotherapies or Radiation Therapies, Targeted or Targeted Radiation Therapies or Radiotherapy, Targeted or Radiotherapies, Targeted or Radiation Therapy, Targeted or Therapies, Targeted Radiation or Targeted Radiotherapy or Therapy, Targeted Radiation).ti,ab. |
| 4 | (biological products or biological product or natural products or natural product or natural agents or natural agent or natural compounds or natural compound).ti,ab,kw. |
| 5 | 3 or 4 |
| 6 | 1 and 2 and 5 |
| 7 | limit 6 to yr="1860 - 2023" |
| 8 | 6 and 7 |

**Scopus (n=96) ：**

| 1 | (TITLE-ABS-KEY("stomatitis" OR "oral mucositis" OR "mouth inflammation") AND TITLE-ABS-KEY("radiotherapy" OR "radiotherapies" OR "radiation therapy" OR "radiation therapies" OR "radiation treatments" OR "radiation treatment") AND TITLE-ABS-KEY("biological products" OR "biological product" OR "natural products" OR "natural product" OR "natural agents" OR "natural agent" OR "natural compounds" OR "natural compound")) |
| --- | --- |

**CINAHL (n=37) ：**

| S1 | stomatitis OR stomatitides OR oral mucositis OR mucositides,oral OR oral mucositidies OR oromucositis OR oromucositides OR mucositis,oral |
| --- | --- |
| S2 | radiotherapy* OR radiation treatment* OR therapy',radiation OR **treatment**,radiation OR radiation therapy* OR targeted radiation therapy'OR radiotherapy',targeted OR radiation therapy',targeted OR targeted radiotherapy' OR therapy',targeted radiation |
| S3 | biologocal products OR medicine, herbal OR biologocal product OR herbal medicine OR herbalism OR traditional medicine practitioners OR phytotherapy OR naturopathy OR Plant Extracts OR Plants, medicinal OR herbal therapy OR herb therapy OR drugs, Chinese herbal OR pharmacognosy OR plant extracts OR ethnopharmacology OR thnobotany OR flower essences OR natural product OR natural products OR natural agent OR natural agents OR natural compound OR natural compounds |
| S4 | S1 AND S2 AND S3 |
